# L-Arginine and Its Metabolites in Age-Related Cerebral Small Vessel Disease with Cognitive Impairment

**DOI:** 10.3390/biom16060914

**Published:** 2026-06-19

**Authors:** Larisa Dobrynina, Alexandra Byrochkina, Kamila Shamtieva, Elena Kremneva, Maryam Zabitova, Alla Shabalina

**Affiliations:** Russian Center of Neurology and Neurosciences, 125367 Moscow, Russia; dobrla@mail.ru (L.D.); byrochkinasasha@mail.ru (A.B.); kamila.shamt@gmail.com (K.S.); moomin10j@mail.ru (E.K.); ashabalina@yandex.ru (A.S.)

**Keywords:** cerebral small vessel disease, L-arginine, subjective cognitive impairment, mild cognitive impairment, diffusion tensor MRI, MRI volumetry, MRI morphometry

## Abstract

A key mechanism in the pathogenesis of cerebral small vessel disease (CSVD) is endothelial dysfunction associated with impaired metabolism of nitric oxide (NO) and its main substrate, L-arginine. The aim of the study was to assess parameters of L-arginine metabolism and their association with MRI-defined brain damage in CSVD patients. A total of 100 CSVD patients (according to MRI STRIVE standards) and cognitive impairment (CI) of varying severity, as well as 20 healthy volunteers, were analyzed. Levels of L-arginine and its metabolites—L-ornithine, L-citrulline, and asymmetric dimethylarginine (ADMA)—were measured; diffusion tensor MRI, MRI volumetry, and morphometry were performed. A threshold level of L-arginine (51.25 μmol/L) was identified, above which an association with CI was observed. Patients with L-arginine ≥ 51.25 μmol/L demonstrated poorer performance on cognitive tests (Stroop test, trail-making test (TMT)-B, TMT B–A, 10-word test) and more severe brain damage, reflected by greater severity of MRI markers (white matter hyperintensities, microbleeds), changes in brain component volumes, cortical atrophy in specific regions, and impairment of white matter microstructural integrity. The obtained data indicate a pathogenetic link between disturbances in L-arginine homeostasis and the development of CSVD with CI and support the need for further studies aimed at refining approaches to their correction.

## 1. Introduction

Age-related cerebral small vessel disease (CSVD) is one of the most common causes of vascular and mixed neurodegenerative cognitive impairment (CI), associated with reduced work capacity and disability in the population [1]. This reflects the importance of its early diagnosis, clarification of the factors and mechanisms underlying its progression, and the development of timely preventive measures.

According to current concepts, the development and progression of CSVD are closely linked to endothelial dysfunction (ED) [2,3,4,5]. Its biological equivalent is a deficiency and/or reduced bioavailability of endothelial nitric oxide (NO). The latter is associated with disruption of vascular wall homeostasis and the development of a vasospastic, prothrombotic, and proinflammatory state of the vascular wall [6], which triggers and sustains the main mechanisms of CSVD, namely hypoxia/ischemia of brain tissue and increased blood–brain barrier (BBB) permeability, followed by neuroinflammation [7,8,9].

NO is synthesized primarily by enzymes of the nitric oxide synthase (NOS) family from the amino acid L-arginine, through its oxidative deamination to L-citrulline with the participation of oxygen, nicotinamide adenine dinucleotide phosphate (NADPH), the cofactor tetrahydrobiopterin (BH_4_), and heme [10,11,12,13]. NO deficiency occurs when L-arginine availability is reduced. The primary mechanisms underlying its reduction include elevated levels of asymmetric dimethylarginine (ADMA), a structural analog of L-arginine that competitively inhibits NOS and thereby reduces NO production, and increased arginase activity. Another highly significant factor contributing to NO deficiency is a lack of exogenous L-arginine or the cofactor BH4, which promotes eNOS uncoupling due to the so-called L-arginine paradox [13,14]. The central principle of this phenomenon is that, although the concentration of endogenous L-arginine (100–800 μM) greatly exceeds the concentrations required to maintain maximal eNOS activity (Km = 3 μM), only exogenous L-arginine is capable of activating eNOS and enhancing NO synthesis [14,15]. In turn, eNOS expression is substantially influenced by systemic pathological processes such as oxidative stress, chronic inflammation, hyperglycemia, hypoxia, and also by insufficient NO production [16,17,18].

Because of its high reactivity, short half-life, and complex metabolism, there are currently no reliable methods for the direct quantitative measurement of NO levels [19]. One of the proposed approaches to assessing its bioavailability is the measurement of blood levels of L-arginine and its closely related amino acid metabolites—L-ornithine, L-citrulline, and ADMA—with the calculation of ratios between them [20,21,22]. The findings for these markers have been inconsistent. Reduced L-arginine/(L-ornithine + L-citrulline) ratio has been associated with coronary artery disease, type 2 diabetes mellitus, and elevated levels of endothelial inflammatory markers VCAM-1 and ICAM-1 [23,24]; a reduced L-arginine/L-ornithine ratio has been linked to an increased 10-year risk of cardiovascular mortality [25]; and a reduced L-arginine/ADMA ratio has been associated with the risk of CSVD in older adults [26]. On the other hand, studies have shown an age-related increase in L-arginine levels [27] and increased hippocampal L-arginine concentrations in the early stages of Alzheimer’s disease and frontotemporal dementia [28]. In studies using the APPswe/PS1ΔE9 mouse model of Alzheimer’s disease, elevated plasma L-arginine levels were detected before the onset of behavioral abnormalities, whereas the development of these abnormalities was associated with increased L-arginine deposition in the prefrontal cortex, hippocampus, and parahippocampal region, as well as with its direct neurotoxic effects [29].

Given the pivotal role of ED in the development of CSVD, investigation of the NO–L-arginine system is highly justified [30]. In the available literature, we did not identify any studies clarifying the associations of circulating L-arginine and its derived indices with the development of CI in CSVD, nor any evidence-based rationale for its clinical use in this context. It should be noted that, despite the pathophysiological rationale for its use across a wide range of diseases associated with ED, the available studies remain limited and have yielded conflicting results. The VINTAGE MI study (Vascular Interaction With Age in Myocardial Infarction, 2002–2004) in post-myocardial infarction patients showed that L-arginine administration at a dose of 9 g/day was associated with higher mortality compared with placebo [31]. At the same time, P. Mone and colleagues (2022), in a cohort of patients with arterial hypertension (AH) and CI, found that 4 weeks of oral L-arginine administration led to improved cognitive performance on the Montreal Cognitive Assessment (MoCA) compared with the placebo group [32]. According to a meta-analysis of 11 randomized clinical trials (*n* = 387), L-arginine administration at a dose of 9 g/day for 4 weeks was associated with a statistically significant reduction in systolic and diastolic blood pressure compared with placebo [33]. Most likely, these opposing therapeutic effects are due to differences in L-arginine metabolism across the studied populations, highlighting the need for criteria to identify patients most likely to benefit from this treatment. In the present study, we examined the associations between structural brain characteristics in patients with CSVD and CI and blood levels of L-arginine and its metabolites in order to identify significant relationships reflecting their biological relevance to disease progression and to help define a target group of patients who may benefit from L-arginine administration.

The aim of this study was to assess markers of L-arginine metabolism and their association with MRI-defined brain damage in patients with CSVD and CI of varying severity.

## 2. Materials and Methods

The study included 100 patients with CSVD (according to MRI STRIVE standards) and CI of varying severity: 80 patients with subjective cognitive impairment (SCI) (62.5 ± 7.1 years; 72.5% women) and 20 patients with mild cognitive impairment (MCI) (65.4 ± 7.3; 65% women). The control group consisted of 20 healthy volunteers (62.6 ± 5.8 years; 55% women) without CI or MRI brain abnormalities. All participants provided written informed consent. The study was approved by the Local Ethics Committee of the Russian Center of Neurology and Neurosciences (protocol No. 10-4/22 dated 23 November 2022).

Exclusion criteria were as follows: CSVD due to other causes; stenotic atherosclerosis of the intra- and/or extracranial arteries > 50%; probable Alzheimer’s disease, defined by an amnestic type of CI and cerebrospinal fluid (CSF) biomarkers; cardiac disease associated with a reduced ejection fraction < 50%; endocrine disorders (type 1 diabetes mellitus (DM), decompensated type 2 DM, or uncontrolled thyroid dysfunction); chronic kidney disease with an estimated glomerular filtration rate < 30 mL/min; absence of somatic diseases explaining the CI; infectious disease within 1 month before blood sampling; and contraindications to MRI.

Clinical assessment included the collection of complaints, medical history, and epidemiological data (sex and age), as well as evaluation of somatic and neurological status and vascular risk factors. Global cognitive function was assessed using the MoCA [34], followed by an extended neuropsychological assessment aimed at evaluating different cognitive domains [35,36,37,38]. Emotional status was assessed using the Hospital Anxiety and Depression Scale (HADS) [39].

All participants underwent structural brain MRI (Siemens MAGNETOM Verio, 3 T and Magnetom Prisma, 3 T, Siemens Healthineers, Erlangen, Germany). The imaging protocol included sequences allowing assessment of CSVD-related MRI markers in accordance with the STRIVE criteria [40]: T2-weighted imaging, 3D T1-MPRAGE, 3D FLAIR, DWI, and SWI in the axial and sagittal planes.

Diffusion tensor MRI (spin-echo echo-planar imaging sequence; b = 0, 1000, and 2500 s/mm^2^; 64 directions; TE/TR 115/12600 ms; resolution 2 × 2 × 2 mm^3^) was performed in 18 patients with CSVD and SCI, 16 patients with CSVD and MCI, and 17 healthy volunteers. Diffusion metric maps, including fractional anisotropy (FA), mean diffusivity (MD), axial diffusivity (AD), and radial diffusivity (RD), were generated in MATLAB R2017a using dedicated scripts [41,42]. Microstructural changes were quantitatively assessed in 29 regions of interest (ROIs), including periventricular, deep, and juxtacortical white matter hyperintensities (WMH), as well as normal-appearing white matter (NAWM) in the frontal and parietal regions, the corpus callosum, cingulate gyrus, uncinate fasciculus, and hippocampus. ROI placement was verified in three planes.

MRI volumetry (T1-MPR; CAT version 12 and SPM version 12 software) of gray matter, white matter, cerebrospinal fluid, and ventricles was performed in 27 patients with CSVD at the SCI stage, 20 patients with CSVD and MCI, and 20 healthy volunteers. The obtained data were normalized to the total intracranial volume (TIV). In addition to visual assessment of cortical thickness, quantitative MRI morphometry of individual cortical regions was performed (T1-MPR; CAT12 and SPM12 software), including voxel-based volumetric analysis (Neuromorphometrics atlas, http://www.neuromorphometrics.com assessed on 19 June 2026) and surface-based cortical thickness analysis (Desikan–Killiany-40 atlas).

Plasma levels of L-arginine and its metabolites—L-ornithine, L-citrulline, and asymmetric dimethylarginine (ADMA)—were measured by high-performance liquid chromatography with tandem mass spectrometry in 80 patients with CSVD and SCI, 20 patients with CSVD and MCI, and 20 healthy volunteers.

Statistical analysis was performed using SPSS Statistics 26.0 software (IBM). Descriptive statistics were used to summarize the data. Categorical variables were compared using the Pearson’s chi-square test or Fisher’s exact test, whereas numeric variables were analyzed using the Mann–Whitney U test or Kruskal–Wallis test followed by pairwise post hoc comparisons with Bonferroni-corrected significance. Data were presented as “Mean ± SD” or Me [25%; 75%].

Associations between variables were assessed using correlation analysis (Spearman’s rank correlation test). Predictive performance was evaluated using binary logistic regression and receiver-operating characteristic (ROC) analysis. Differences were considered significant at *p* < 0.05.

## 3. Results

The patient groups and controls were comparable in terms of sex, age, and level of education (Table 1).

Among the assessed vascular risk factors, patients with CSVD showed a statistically significant predominance of AH, DM, and hypercholesterolemia compared with controls. At the same time, no statistically significant differences in the frequency of these vascular risk factors were found between the SCI and MCI groups.

**Figure 1:**
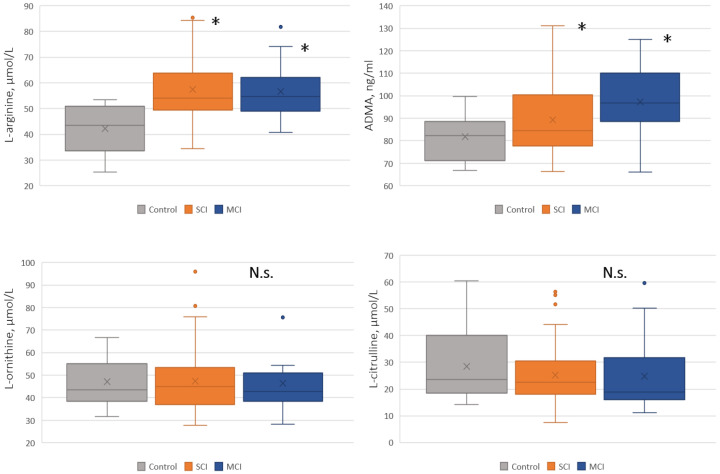
Levels of L-arginine, L-citrulline, L-ornithine, and ADMA in patients with CSVD (n = 100) and in the control group (n = 20). Notes: * *p* < 0.05 vs. control; N.s.—non-significant. SCI—subjective cognitive impairments, MCI—mild cognitive impairment, ADMA—asymmetric dimethylarginine.

Blood levels of L-arginine and ADMA were higher in both the SCI and MCI groups than in controls, with no significant difference between the two patient groups (Figure 1).

**Figure 2:**
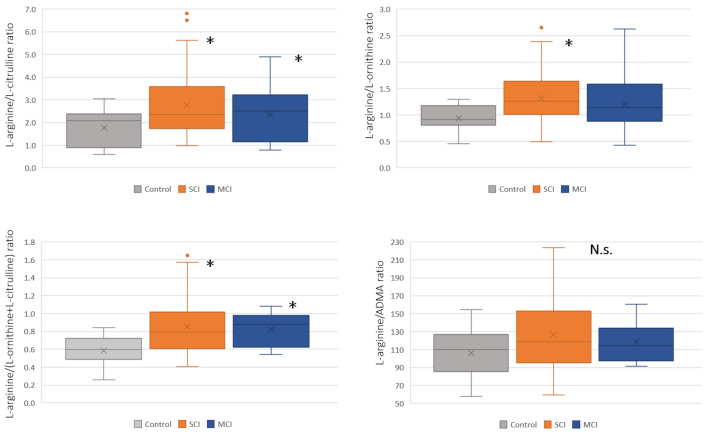
Calculated ratios of L-arginine to its amino acid metabolites in patients with CSVD (n = 100) and in the control group (n = 20). Notes: * *p* < 0.05 vs. control; N.s.—non-significant. SCI—subjective cognitive impairments, MCI—mild cognitive impairment, ADMA—asymmetric dimethylarginine.

L-arginine levels did not differ between patients with CSVD at the SCI and MCI stages but were significantly higher than those in the control group. ADMA levels increased with increasing severity of the CI and differed significantly from those in the control group at each stage, but not between the CI severity groups. The calculated ratios of L-arginine to its amino acid metabolites, except for ADMA, were significantly higher in patients with SCI and MCI than in controls, with no difference between the two patient groups (Figure 2).

**Figure 3:**
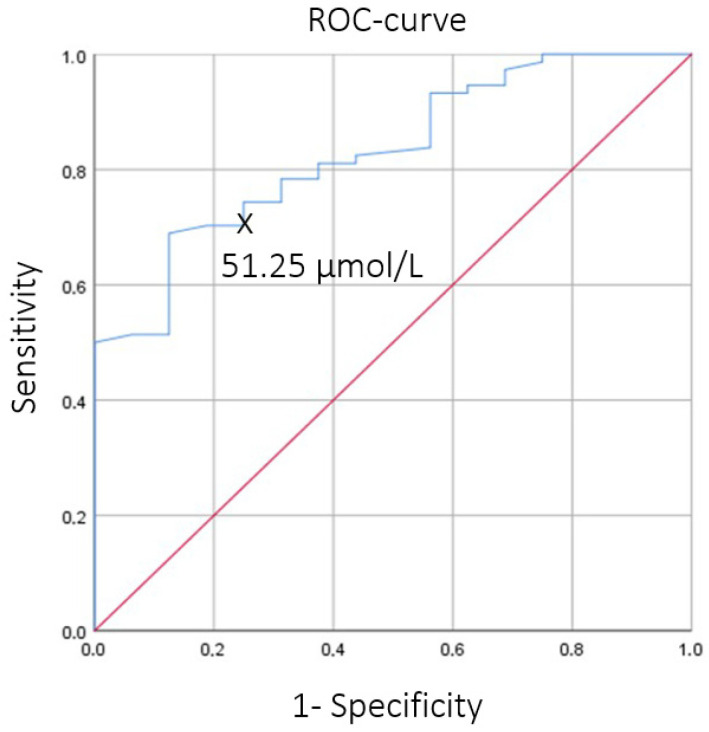
ROC curve of L-arginine for SCI in CSVD. Note: SCI—subjective cognitive impairments, CSVD—cerebral small vessel disease, the blue ROC curve, the red reference line.

In addition, logistic regression adjusted for age and sex was used to assess the effect of L-arginine on the development of early CSVD with SCI. L-arginine remained an independent statistically significant predictor after inclusion of these covariates in the model (for age, sex, and L-arginine). The resulting model demonstrated high predictive performance in ROC analysis with an area under the curve (AUC) of 0.889 (95% CI 0.800–0.977; *p* < 0.05). When L-arginine was evaluated separately using ROC analysis, AUC was 0.83 (95% CI 0.70–0.90; *p* < 0.05), indicating its independent predictive value for early CSVD with SCI (Figure 3).

ROC analysis identified an L-arginine cutoff value of 51.25 μmol/L (sensitivity 68.9% and specificity 87.5%). Based on this threshold, patients with SCI were stratified into groups above and below this cutoff value. Comparison of these two groups revealed differences in macrostructural MRI markers of CSVD and in cognitive test performance (Table 2 and Table 3). There was no MRI evidence of acute or subacute recent small subcortical infarct in the patient groups.

Patients with L-arginine levels above the cutoff value had a greater number of cerebral microbleeds and tended to have more severe white matter hyperintensities (*p* = 0.062).

Comparison of cognitive test results showed that the group with L-arginine levels > 51.25 μmol/L had statistically significantly poorer performance on the Stroop test (interference), TMT-B, TMT-A, TMT B–A, and the 10-word test (trial 5 and delayed recall). The studied patient groups did not differ significantly in anxiety and depression scores or in vascular risk factors.

Further analysis of the relationships between MRI volumetric and morphometric measures and L-arginine levels, as well as their calculated ratios, revealed a statistically significant deterioration of the assessed brain parameters with increasing L-arginine levels (Table 4).

Statistically significant weak associations were identified between higher blood L-arginine levels and lower total brain volume, total gray matter volume, and the volumes of individual cortical regions, as well as greater cerebrospinal fluid volume. Higher blood ADMA levels and higher L-arginine/(L-ornithine + L-citrulline) ratio were associated with lower total gray matter volume, whereas higher ADMA levels and higher L-arginine/(L-ornithine + L-citrulline) and L-arginine/ADMA ratios were associated with atrophy in certain cortical regions.

**Figure 4:**
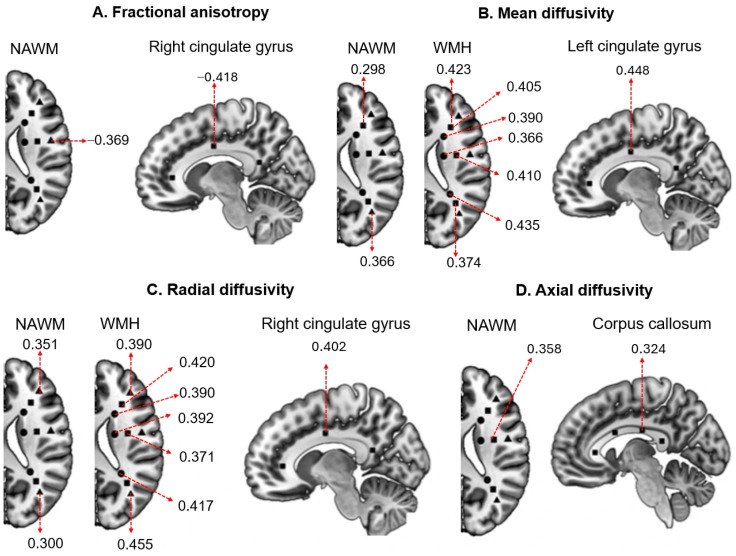
Associations between L-arginine levels and diffusion tensor MRI metrics in specific brain regions (ROIs): (**A**), fractional anisotropy (FA); (**B**), mean diffusivity (MD); (**C**), radial diffusivity (RD); (**D**), axial diffusivity (AD) in specific brain regions. Note: Values represent Spearman’s rank correlation coefficients (r_s_). NAWM — normal-appearing white matter; WMH — white matter hyperintensity. ▲—juxtacortical white matter, ■—deep white matter, ●—periventricular white matter.

Correlation analysis showed that elevated L-arginine levels were associated with signs of white matter microstructural damage. Inverse correlations were observed with fractional anisotropy (FA) in the frontal regions and cingulate gyrus (r = −0.4), as well as direct correlations with mean diffusivity (MD) and radial diffusivity (RD), predominantly in the frontal and temporoparietal regions and the cingulate gyrus (r = 0.3–0.5). Associations with axial diffusivity (AD) were less pronounced (r = 0.3–0.4) and were found in the posterior frontal lobe and corpus callosum (Figure 4A–D, Supplementary, Tables S1 and S2).

The derived L-arginine ratios − L-arginine/(L-ornithine + L-citrulline), L-arginine/L-ornithine, and L-arginine/L-citrulline − showed more widespread and pronounced associations. For FA, inverse correlations were observed predominantly in the frontal regions, temporoparietal area, and corpus callosum (r = −0.3 to −0.4); for MD and RD, direct correlations were found in the same regions, including the cingulate gyrus and corpus callosum (r = 0.3–0.5); for AD, the most pronounced correlations were also observed in the posterior frontal lobe (r = 0.3–0.5). The L-arginine/ADMA ratio showed a limited number of associations, mainly with RD values in the frontal and temporoparietal regions (r = 0.34) (Supplementary, Tables S3 and S4).

## 4. Discussion

The basis for this study was the need to identify markers associated with the mechanisms of CSVD and its progression. The main contradiction of age-related CSVD is that, despite their close association with classic vascular risk factors, there is no direct cause-and-effect relationship between them.

The central role of ED and the associated reduction in NO bioavailability in the initiation and progression of CSVD [2,3,4,5], together with the dependence of NO synthesis on L-arginine and its metabolites [10,11,12], explain the choice of these compounds for study. The study design included assessment of blood levels of L-arginine and its metabolites in patients with CSVD and CI of varying severity, with calculation of several derived ratios, and comparison of these measures with diagnostic MRI markers of CSVD, microstructural changes in WMH and NAWM, brain volumetric measures, and different cortical regions.

In our cohort, patients with CSVD had significantly higher L-arginine levels than healthy volunteers. This initially appeared counterintuitive and prompted us to review studies reporting relative L-arginine levels. L-arginine levels show considerable variability in reference values across studies [43,44,45], although an age-related increase has been consistently reported [27]. Given that aging is associated with impaired endothelial function and, accordingly, reduced NO bioavailability, one possible explanation for this phenomenon is an age-related disturbance of L-arginine metabolism, resulting in the accumulation of endogenous L-arginine as an end product.

Our comparison of L-arginine levels and derived ratios involving its metabolites across CSVD groups with different severities of CI (SCI and MCI) and healthy controls showed statistically significant differences versus controls, but no differences between the groups with different CI severity.

For the subsequent analyses, we focused on the SCI group, as these patients had less pronounced brain changes than those with MCI and therefore allowed assessment of earlier disease stages. We found that L-arginine itself was highly significant (*p* < 0.001), and we identified a cutoff value of 51.25 μmol/L, above which L-arginine was associated with the development of CSVD with SCI. To further clarify the associations between elevated L-arginine and brain injury, we divided patients with SCI into groups with L-arginine levels of ≥51.25 and <51.25 μmol/L. Patients with blood L-arginine levels ≥ 51.25 μmol/L showed significantly poorer performance on tests of executive function and memory, as well as a greater burden of cerebral microbleeds and WMH. Higher L-arginine levels were also associated with impaired brain microstructural integrity on diffusion tensor MRI, reflected by reduced fractional anisotropy (FA) and increased mean diffusivity (MD), radial diffusivity (RD), and axial diffusivity (AD) in different regions of WMH and NAWM, as well as with greater brain damage based on volumetric measures of the whole brain and individual regions.

These findings may suggest more severe brain damage in patients with L-arginine levels ≥ 51.25 μmol/L. It is possible that elevated circulating L-arginine levels are associated with the accumulation of endogenous L-arginine and its potentially toxic effects on the brain. It should be emphasized that no differences were found between the groups with L-arginine levels above and below the cutoff value in the prevalence of classical vascular risk factors (AH, DM, smoking, and obesity) or in the severity of emotional disturbances (anxiety and depression).

The possibility of a direct toxic effect of elevated L-arginine levels on the brain is demonstrated by both experimental and clinical studies. Thus, in a mouse model of Alzheimer’s disease (APPswe/PS1ΔE9), elevated plasma L-arginine levels preceded the onset of behavioral abnormalities and coincided with increased L-arginine accumulation in the prefrontal cortex, hippocampus, and parahippocampal region [29]. Similar findings have also been reported in humans: patients with early Alzheimer’s disease and frontotemporal dementia were found to have increased hippocampal L-arginine levels compared with controls [28]. Indirect support for this concept also comes from emerging therapeutic strategies for certain cancers based on L-arginine depletion/deprivation in so-called “arginine-dependent cancers” [46,47].

As mentioned above, the main mechanisms underlying the accumulation of endogenous L-arginine with potential toxic effects are increased ADMA levels [48,49,50] and reduced arginase activity [51]. In our study, similarly to other reports [26,52,53], a significant increase in ADMA levels was observed in CSVD. ADMA is a competitive inhibitor of eNOS, which is associated with reduced NO synthesis and, consequently, the development of ED, as well as the emergence of the “L-arginine paradox,” that is, dependence on exogenous L-arginine despite high levels of endogenous L-arginine. A second key mechanism that may lead to elevated L-arginine levels is reduced arginase activity. This enzyme, which participates in the urea cycle, competes with eNOS for the substrate and thereby regulates NO synthesis [14]. Arginase deficiency, particularly in endothelial cells, disrupts L-arginine metabolism, leading to ED, inflammation, and increased production of reactive oxygen species [20,54]. This is further supported by the results of a recent study using a human brain capillary endothelial cell model (hCMEC/D3), in which arginase deficiency led to L-arginine accumulation, increased NO levels, and oxidative stress, thereby suppressing cell viability [51].

## 5. Conclusions

This study assessed blood levels of L-arginine and amino acids closely related to its metabolism in age-related CSVD and identified an L-arginine cutoff value associated with an increased risk of early CI. Higher L-arginine levels were also associated with more severe brain damage, reflected by greater severity of MRI markers (WMH and cerebral microbleeds), changes in brain structure (including brain volume, gray matter volume, cerebrospinal fluid volume, and individual cortical regions), and impaired microstructural integrity in both affected and NAWM. The identified associations between elevated L-arginine levels, CI, and brain damage support the need for further studies aimed at identifying ways to overcome these disturbances in L-arginine metabolism in CSVD, given its crucial role in NO synthesis and, consequently, in endothelial biological responses.

## 6. Limitations of the Study

The study focused on assessing L-arginine and its metabolites and did not include analysis or clarification of relationships with other circulating biomarkers associated with the mechanisms of CSVD development. Going forward, it seems appropriate to compare L-arginine metabolism indicators with recognized markers of ED, as well as with neuroimaging indicators of white matter microstructural integrity and BBB permeability. This will allow for a more comprehensive assessment of the role of L-arginine metabolism disorders in the development and progression of CSVD and associated CI.

## Figures and Tables

**Table 1:** Clinical and demographic characteristics of the study groups.

Parameters	CSVD			*p*
	SCI n = 80	MCI n = 20	Control n = 20	
**Age, years**	62.5 ± 7.1	65.4 ± 7.3	62.6 ± 5.8	n.s.
**Gender:** ▪ Women, (n, %)	58 (72.5%)	13 (65%)	11 (55%)	n.s
▪ Men, (n, %)	22 (27.5%)	7 (35%)	9 (45%)	
**Risk factors, n (%):**		(n, %)		
▪ Arterial hypertension	80 (100%)	20 (100%)	8 (40%)	*
▪ Diabetes mellitus type 2	8 (10%)	6 (30%)	1 (5%)	n.s
▪ Obesity (body mass index > 30 kg/m^2^)	34 (42.5%)	7 (35%)	5 (25%)	n.s
▪ Hypercholesterolemia	58 (72.5%)	15 (75%)	5 (25%)	*
▪ Smoking	13 (16.25%)	3 (15%)	1 (5%)	n.s
**General level of education, years**	14.5 ± 3.4	14.5 ± 2.9	16.6 ± 2.8	n.s

Note: * *p* < 0.05; n.s.—non-significant. The *p*-values correspond to the overall comparison across the three study groups.

**Table 2:** Comparison of macrostructural MRI markers of CSVD between patient groups with CSVD and SCI above and below the L-arginine cutoff value.

MRI Markers of CSVD	L-Arginine <51.25 μmol/L n = 26	L-Arginine ≥51.25 μmol/L n = 54	*p*
Lacunes, (n, %)	8 (10%)	22 (27.5%)	n.s.
Microbleeds, (n, %)	3 (3.75%)	19 (23.75%)	*
White matter hyperintensity, Fazekas score (F), (n, %)			
▪ F1	11 (13.75%)	13 (16.25%)	
▪ F2	12 (15%)	19 (23.75%)	n.s.
▪ F3	3 (3.75%)	22 (27.5%)	

Note: * *p* < 0.05; n.s.—non-significant.

**Table 3:** Comparison of cognitive test results between patient groups with SCI in CSVD and L-arginine levels above and below the cutoff value.

Parameters	L-Arginine <51.25 μmol/L, n = 26	L-Arginine ≥51.25 μmol/L, n = 54	*p*
TMT A (s)	40 [30; 61]	50 [40; 64]	*
TMT B (s)	85 [73; 115]	131 [92; 191]	*
TMT B-A (s)	37 [25; 68]	77 [45; 138]	*
Stroop test, interference (s)	126 [103; 138]	150 [130; 180]	*
10-word test, delayed recall (number of words)	9 [7; 10]	7 [6; 8]	*
MoCA	28 [27; 29]	28 [27; 28]	n.s.

Note: * *p* < 0.05; n.s.—non-significant.

**Table 4:** Associations of markers of L-arginine metabolism with neuroimaging volumetric and morphometric measures.

Parameters	L-Arginine	ADMA	L-Arginine/ L-Ornithine + L-Citrulline	L-Arginine/ ADMA
Volumetric measures:				
TBV/TIV	−0.342	n.s.	n.s.	n.s.
GM/TIV	−0.388	−0.332	−0.326	n.s.
CSF/TIV	0.310	n.s.	n.s.	n.s.
Volumes of individual cortical regions:				
Angular gyrus, R	−0.301	n.s.	n.s.	n.s.
Operculum insulae, R	−0.361	n.s.	n.s.	−0.313
Middle temporal gyrus, R	−0.303	n.s.	n.s.	n.s.
Middle temporal gyrus, L	n.s.	−0.303	n.s.	n.s.
Precentral gyrus, R	−0.356	n.s.	n.s.	n.s.
Precentral gyrus, L	−0.350	n.s.	n.s.	n.s.
Isthmus of cingulate gyrus, L	n.s.	n.s.	−0.336	n.s.
Isthmus of cingulate gyrus, R	n.s.	n.s.	−0.300	n.s.
Thickness of individual cortical regions:				
Paracentral lobule, R	n.s.	−0.303	n.s.	n.s.
Insula, L	n.s.	−0.341	n.s.	n.s.

Note: values represent Spearman’s rank correlation coefficients (r_s_); n.s.—non-significant. TBV—Total Brain Volume, GM—Gray Matter, CSF—cerebrospinal fluid, TIV—Total Intracranial Volume, R—right hemisphere of the cerebrum, L—left hemisphere of the cerebrum.

## Data Availability

The data presented in this study are available upon reasonable request from the corresponding author.

## Supplementary Materials

The following supporting information can be downloaded at https://www.mdpi.com/article/10.3390/biom16060914/s1. Table S1: Associations between blood levels and derived ratios of L-arginine metabolism markers and fractional anisotropy (FA) among study participants. Table S2: Associations between blood levels and derived ratios of L-arginine metabolism markers and mean diffusivity (MD) values among study participants. Table S3: Associations between blood levels and derived ratios of L-arginine metabolism markers and radial diffusivity (RD) values among study participants. Table S4: Associations between blood levels and derived ratios of L-arginine metabolism markers and axial diffusivity (AD) val-ues among study participants.

## Abbreviations

The following abbreviations are used in this manuscript:

CSVD	Cerebral Small Vessel Disease
CI	Cognitive Impairments
SCI	Subjective Cognitive Impairments
MCI	Mild Cognitive Impairments
ADMA	Asymmetric dimethylarginine
ED	Endothelial dysfunction
NO	Nitric oxide
NAWM	Normal appearing white matter
WMH	White matter hyperintensities
