# Supplementary material for: L-Arginine and Its Metabolites in Age-Related Cerebral Small Vessel Disease with Cognitive Impairment
Source: Biomolecules. 2026 Jun 19;16(6):914. doi: 10.3390/biom16060914 (PMC13297444; doi:10.3390/biom16060914)
Supplement: Supplementary file 1 [file biomolecules-16-00914-s001.zip › biomolecules-4320042-supplementary.pdf]

Supplementary Appendix 1

Table S1. Associations between blood levels and derived ratios of L-arginine metabolism markers and fractional anisotropy (FA) among study participants.

FA in ROIs	L-arginine	L-arginine/ L-ornithine + L-citrulline	L-arginine/ L-ornithine	L-arginine/ L-citrulline
Frontal lobe, posterior parts				
jNAWM	-0.369	-0.339	-0.326	-0.320
pNAWM	n.s.	n.s.	n.s.	-0.333
jWMH	n.s.	n.s.	n.s.	-0.442
Tempo-pariental lobe				
jNAWM	n.s.	-0.331	-0.304	-0.313
Cingulate gyrus, right				
middle parts	-0.418	-0.366	-0.338	-0.312
Corpus callosum				
anterior parts	n.s.	n.s.	n.s.	-0.365
anterior-middle parts	n.s.	-0.393	-0.306	-0.313
Hippocampus				
right	n.s.	n.s.	-0.315	n.s.

Note. Values represent Spearman's rank correlation coefficients (r_s); n.s. — non significant.
FA, fractional anisotropy (×10^-2^); jNAWM, juxtacortical normal-appearing white matter; dNAWM, deep normal-appearing white matter; pNAWM, periventricular normal-appearing white matter; jWMH, juxtacortical white matter hyperintensity; ROIs, regions of interest.

Table S2. Associations between blood levels and derived ratios of L-arginine metabolism markers and mean diffusivity (MD) values among study participants.

MD in ROIs	L-arginine	L-arginine/ L-ornithine + L-citrulline	L-arginine/ L-ornithine	L-arginine/ L-citrulline	L-arginine/ ADMA
Frontal lobe, anterior parts					
jNAWM	n.s.	0.319	n.s.	n.s.	n.s.
dNAWM	0.298	0.365	n.s.	0.434	n.s.
pWMH	0.405	0.436	0.345	0.386	n.s.
dWMH	0.423	n.s.	n.s.	n.s.	n.s.
Frontal lobe, posterior parts					
dNAWM	n.s.	0.449	0.325	n.s.	n.s.
pWMH	0.366	n.s.	n.s.	n.s.	n.s.
dWMH	0.410	0.358	0.368	n.s.	n.s.
Tempo-pariental lobe					
jNAWM	0.366	0.359	n.s.	0.318	n.s.
jWMH	n.s.	0.382	n.s.	n.s.	n.s.
pWMH	0.435	0.527	0.518	0.340	n.s.
dWMH	0.374	0.452	0.405	n.s.	0.310
Cingulate gyrus, left					

anterior parts	n.s.	0.319	n.s.	0.340	n.s.
middle parts	0.448	0.456	0.355	0.357	0.319
Corpus callosum					
anterior-middle parts	n.s.	0.316	n.s.	n.s.	n.s.

Note. Values represent Spearman's rank correlation coefficients (r_s); n.s. — non significant.
MD, mean diffusivity (mm²/s × 10^{-4}); jNAWM, juxtacortical normal-appearing white matter; dNAWM, deep normal-appearing white matter; pNAWM, periventricular normal-appearing white matter; jWMH, juxtacortical white matter hyperintensity; dWMH, deep white matter hyperintensity; pWMH, periventricular white matter hyperintensity; ROIs, regions of interest.

Table S3. Associations between blood levels and derived ratios of L-arginine metabolism markers and radial diffusivity (RD) values among study participants.

RD in ROIs	L-arginine	L-arginine/ L-ornithine + L-citrulline	L-arginine/ L-ornithine	L-arginine/ L-citrulline	L-arginine/ ADMA
Frontal lobe, anterior parts					
jNAWM	n.s.	0.335	n.s.	0.340	n.s.
dNAWM	n.s.	0.354	n.s.	0.448	n.s.
jWMH	0.390	n.s.	n.s.	n.s.	n.s.
pWMH	0.390	0.410	0.337	n.s.	n.s.
dWMH	0.420	n.s.	n.s.	n.s.	0.344
Frontal lobe, posterior parts					
jNAWM	0.351	0.320	0.311	n.s.	n.s.
pWMH	0.371	n.s.	n.s.	n.s.	n.s.
dWMH	0.392	0.335	0.359	n.s.	n.s.
Tempo-pariental lobe					
jNAWM	0.300	0.480	0.366	0.421	n.s.
jWMH	n.s.	0.395	n.s.	n.s.	n.s.
pWMH	0.417	0.523	0.508	0.351	n.s.
dWMH	0.455	0.531	0.471	0.331	0.342
Cingulate gyrus, right					
middle parts	0.402	0.336	0.324	0.301	n.s.
posterior parts	0.322	0.348	0.390	n.s.	n.s.
Cingulate gyrus, left					
middle parts	n.s.	0.372	n.s.	n.s.	n.s.
Corpus callosum					
anterior-middle parts	n.s.	0.369	0.300	n.s.	n.s.

Note. Values represent Spearman's rank correlation coefficients (r_s); n.s. — non significant
RD, radial diffusivity (mm²/s × 10^{-4}); jNAWM, juxtacortical normal-appearing white matter; dNAWM, deep normal-appearing white matter; pNAWM, periventricular normal-appearing white matter; jWMH, juxtacortical white matter hyperintensity; dWMH, deep white matter hyperintensity; pWMH, periventricular white matter hyperintensity; ROIs, regions of interest.

Table S4. Associations between blood levels and derived ratios of L-arginine metabolism markers and axial diffusivity (AD) values among study participants.

AD in ROIs	L-arginine	L-arginine/ L-ornithine + L-citrulline	L-arginine/ L-ornithine	L-arginine/ L-citrulline
Frontal lobe, posterior parts				
dNAWM	0.358	0.502	0.349	0.541
Corpus callosum				
posterior-middle parts	0.324	n.s.	n.s.	n.s.
Uncinate fasciculus				
left	0.324	0.346	n.s	0.335

Note. Values represent Spearman's rank correlation coefficients (r_s); n.s. — non significant

AD, axial diffusivity (mm²/s × 10^{-4}); dNAWM, deep normal-appearing white matter; ROIs, regions of interest.

https://doi.org/10.3390/xxxxx
